# Identification of *BBX* gene family and its function in the regulation of microtuber formation in yam

**DOI:** 10.1186/s12864-023-09406-1

**Published:** 2023-06-26

**Authors:** Yingying Chang, Haoyuan Sun, Shiyu Liu, Yulong He, Shanshan Zhao, Jiage Wang, Tianle Wang, Jiangli Zhang, Jin Gao, Qingxiang Yang, Mingjun Li, Xiting Zhao

**Affiliations:** 1 College of Life Sciences, Henan Normal University, Xinxiang, 453007 China; 2 Engineering Technology Research Center of Nursing and Utilization of Genuine Chinese Crude Drugs in Henan Province / Engineering Laboratory of Green Medicinal Material Biotechnology of Henan Province, Xinxiang, 453007 China; 3 Henan International Joint Laboratory of Agricultural Microbial Ecology and Technology, Xinxiang, 453007 China

**Keywords:** Yam, *BBX* gene family, Identification, Function, *DoBBX2/DoCOL5*, *DoBBX8/DoCOL8*, Microtuber formation

## Abstract

BBX proteins play important roles in all of the major light-regulated developmental processes. However, no systematic analysis of *BBX* gene family regarding the regulation of photoperiodic microtuber formation has been previously performed in yam. In this study, a systematic analysis on the *BBX* gene family was conducted in three yam species, with the results, indicating that this gene plays a role in regulating photoperiodic microtuber formation. These analyses included identification the *BBX* gene family in three yam species, their evolutionary relationships, conserved domains, motifs, gene structure, *cis*-acting elements, and expressional patterns. Based on these analyses, *DoBBX2/DoCOL5* and *DoBBX8/DoCOL8* showing the most opposite pattern of expression during microtuber formation were selected as candidate genes for further investigation. Gene expression analysis showed *DoBBX2/DoCOL5* and *DoBBX8/DoCOL8* were highest expressed in leaves and exhibited photoperiod responsive expression patterns. Besides, the overexpression of *DoBBX2/DoCOL5* and *DoBBX8/DoCOL8* in potato accelerated tuber formation under short-day (SD) conditions, whereas only the overexpression of *DoBBX8/DoCOL8* enhanced the accelerating effect of dark conditions on tuber induction. Tuber number was increased in *DoBBX8/DoCOL8* overexpressing plants under dark, as well as in *DoBBX2/DoCOL5* overexpressing plants under SD. Overall, the data generated in this study may form the basis of future functional characterizations of *BBX* genes in yam, especially regarding their regulation of microtuber formation *via* the photoperiodic response pathway.

**Supplementary Information:**

The online version contains supplementary material available at 10.1186/s12864-023-09406-1.

## Introduction

Yam (*Dioscorea* spp.) is an important tuberous crop belonging to the Dioscoreaceae family, grown in various regions globally [1, 2]. *D. opposita* ‘Tiegun’ is a popular Chinese yam cultivar that has been commercially grown in Jiaozuo city of Henan province, China for a long time due to its medicinal and nutritional benefits [3]. However, vegetative propagation of yam through its tubers year after year leads to the spread of viral infections and declining quality [4–6]. Researchers have successfully developed microtuber culture techniques, providing virus-free tubers suitable for transportation and storage [7].

Tuberization in yam [8] and other tuberous crops [9, 10] may involve hormomes (e.g., gibberellins (GAs) and abscisic acid (ABA)) and genes in GA and ABA synthesis, catabolism, and signalling [11–13]. Tuberization in model plant species, potato (*Solanum tuberosum*) [14] may also be regulated by photoperiodic genes such as circadian-regulated gene *CONSTANS* (CO) and the CO/FT module [15, 16]. In potato, StCO inhibited the transcription of the tuberigen StSP6A in leaves and repressed tuberization in a photoperiod-dependent manner. However, it is unknown whether tuberization in yam involves photoperiod-dependent genes, such as the *BBX* family—ZFP transcription factors.

The BBX family, also known as CONSTANS-LIKE (COL) proteins, are important in photomorphogenesis, seed germination, shade avoidance, photoperiodic regulation of flowering and tuberization [14, 17]. In *Arabidopsis*, the *BBX* gene family has 32 members divided into five structural groups based on their domains [18]. *AtBBXs* are mainly involved in the regulation of flowering. It has been reported that some *BBX* genes function to regulate flowering and are also involved in the regulation of tuberization. For example, *AtBBX1/AtCO* promotes flowering under LD conditions, but inhibites tuber development under SD conditions [19, 20]. *AtBBX6/COL5* accelerates flowering [21], while over-expression of *COL5* from lotus (*Nelumbo nucifera*) in potato increased tuber weight, but without changing the number of tubers under SD conditions [22]. Besides, *AtBBX21* promoting seedling photomorphogenesis, also increased tuber yield in potato [23].

We obtained genomes and transcriptomes of the white Guinea yam (*D. rotundata*), the greater yam (*D. alata*), and *D. opposita* ‘Tiegun’, and analyzed the *BBX* gene family in the species with a focus on physical and chemical properties, conserved domains, gene structure, chromosome distribution, collinearity, and *cis*-acting elements. We identified two candidate *DoBBX* genes in ‘Tiegun’, and investigated their expression patterns in different tissues, under diurnal cycle and continuous light, and at five tuberization stages. The function of *DoBBX2/DoCOL5* and *DoBBX8/DoCOL8* in regulation of tuberization in potato was identified through heterologous overexpression. This study provides insights into the *BBX* gene family and the function of *DoBBX*s in microtuber formation.

## Materials and methods

### Genome and transcriptome resources

Chromosome-level reference genomes of the white Guinea yam (*D. rotundata*) [24], and the greater yam (*D. alata*) [25], *A. thaliana* version 10 (TAIR 10) [26] and *Oryza sativa* version 7.0 [27] were downloaded from https://genome-e.ibrc.or.jp/home/bioinformatics-team/yam, https://genome.jgi.doe.gov/info/Dalata_v2_1, http://www.arabidopsis.org, and https://genome.jgi.doe.gov/portal/pages/dynamicOrganismDownload.jsf?organism=Osativa, respectively. Our previous transcriptome data were deposited in NCBI Sequence Read Archive (SRA, http://www.ncbi.nlm.nih.gov/Traces/sra) with accession number SRP061414 [8].

### Identification of the *BBX* gene family in yam

To identify the yam *BBX* genes, the B-box domain (Pfam00643/cl00034) was downloaded from the PFAM database (https://pfam.xfam.org/) [28] and was used as the query sequence to find the predicted proteins in yam using the HMMER 3.0 program with the threshold of E-value < 10^–20^ [29]. The predicted proteins containing the B-box domain were used to construct a yam-specific HMM file via hmmbuild from HMMER 3.0 [29]. The yam-specific BBX HMM was used as query against the predicted proteins of yam. The peptide sequences with the threshold of E-value < 10^–10^ and containing the B-box domain identified by the PFAM database [28] and NCBI-CDD tools (https://www.ncbi.nlm.nih.gov/Structure/bwrpsb/bwrpsb.cgi) [30] were selected as candidate proteins. The ClustalW program in MEGA 7.0 and chromosomal locations of candidate genes learned from GFF3 files were used to remove the repetitive sequences and the redundant alternatively spliced sequences [31]. The IDs, characteristics and sequences of BBX proteins from three yam species were listed in Supplementary Tables 1–3, and were named according to their relationships with homologous genes in *A. thaliana*.

### Multiple sequence alignment and phylogenetic analyses of *BBX* gene family in yam

All the amino acid residues of *DoBBXs*, *DrBBXs*, *DaBBXs, AtBBXs *and *OsBBXs* were aligned by MEGA 7.0 [31], and the phylogenetic tree was constructed *via* the neighbor-joining (NJ) method with 1000 bootstrap replicates.

### Chromosomal distribution and *gene* duplications

The lengths of the chromosomes and the physical locations of *DrBBX*s and *DaBBX*s were obtained from the genome annotation information (gff3) of *D. rotundata* and *D. alata*, respectively. MapGene2Chromosome v2 software (http://mg2c.iask.in/mg2c_v2.0/) was used to map the distribution of *DrBBX* and *DaBBX* genes [32]. MCScanX software was used to detect the genome replication gene pairs in *D. alata* and between different species [33]. Nucleotide sequences with alignment ratios and similarity ratios greater than 75% and with distances between genes on the same chromosome of less than 100 kb were selected as tandem duplications. Moreover, the genes located in the duplicated regions and nucleotide sequences with alignment ratios greater than 75% were selected as resulting from segmental duplications [34]. Tbtools was used to analyze collinearity of the *BBX* genes and to visualize the duplicated gene pairs [35, 36]. The KaKs_Calculator1.2 software was used to calculate the nonsynonymous (*Ka*), synonymous (*Ks*) substitution rates, and *Ka/Ks* values of the different gene duplication pairs [37]. The *Ks* values were used to estimate the approximate date of every duplicated event occurred in yam, using the formula: T = *Ks*/2λ × 10^–6^ Mya (λ = 6.5 × 10^−9^) (Supplementary Table 4).

### Protein domains, motifs and gene structure analyses of *DrBBXs* and *DaBBXs*

The sequence logos of conserved domains of B-box1, B-box2 and CCT were generated by WebLogo (*Web-based sequence logo generating application; *Weblogo.berkeley.edu) [38, 39]. EvolView [40, 41] (https://www.evolgenius.info/evolview/#login) was used for phylogenetic tree visualization and conserved domain annotations. The conserved motifs of DrBBXs and DaBBXs proteins were identified by the MEME v4.9.0 (http://meme-suite.org/tools/meme) with the optimum motifs set at ≥ 10 and ≤ 50 amino acids and the maximum number of motifs set at 10 [42]. Gene Structure Display Server (http://gsds.cbi.pku.edu.cn/) was used to analyze the intron–exon distribution from the CDS and genomic sequence files of *DrBBX*s and *DaBBX*s [43].

### *Cis*-acting elements in the promoter regions of *DrBBXs* and *DaBBXs*

We considered the sequence 2000 bp upstream of the initiation codons as the proximal promoter region sequences. Promoters were predicted in the PlantCARE database (http://bioinformatics.psb.ugent.be/webtools/plantcare/html/) [44] and were categorized for functional groups and visualized using EvolView [40, 41].

### Excavation of the differentially expressed *BBX* genes during microtuber formation in *D. opposita* ‘Tiegun’

The transcriptome sequencing datasets during tuberization of a traditional Chinese medicinal plant *D. opposita* ‘Tiegun’ were retrieved form the NCBI Sequence Read Archive (SRA) repository (http://www.ncbi.nlm.nih.gov/sra?term=SRP061414). The transcriptome data was processed following Kim et al. (2018) [45]. In brief, the SRA files were converted into Fastqa format using the SRAToolkit software [46]. The raw reads were then subjected to quality control using Trimmomatic and FastQC softwares [47, 48]. Subsequently, high-quality reads were aligned to the reference genome from *D. alata* using the HISAT2 (version: 2.0.5). The mapped reads of were assembled and quantified using the StringTie program [49]. The FPKM was obtained for further analysis. The expression patterns of *DoBBX* family members during tuberization were examined using the FPKM and the expression heatmap was generated using the pheatmap R package [50].

### Plant materials and growth condition

*D. opposita* ‘Tiegun’ were cultured on the MS medium containing 3 g·L^−1^ agar and 30 g·L^−1^sucrose in Engineering Technology Research Center of Nursing and Utilization of Genuine Chinese Crude Drugs in Henan Province (Henan Normal University in Xinxiang, China). The growth conditions were kept at 23 ± 2 °C with 16 h light/8 h dark photoperiod under 38 μm·sec^−1^·m^−2^ light intensity, with medium change every four weeks. For microbuer induction, plants from four-week-old *D. opposita* ‘Tiegun’ were grown on MS medium containing and 60 g·L^−1^sucrose with shaking (120 r·m^−1^) at 23 ± 2 °C under dark.

A Chinese potato variety ‘E-potato-3’ (*S. tuberosum* ‘E3’) kindly provided by Huazhong Agricultural University was used for genetic transformation. The potato seedlings were cultured on the potato medium (P0: MS + 30 g·L^−1^ sucrose + 3 g·L^−1^ agar) under the following: the growth conditions 23 ± 2 °C with 16 h light/8 h dark photoperiod (long day condition, LD) under 38 μm·sec^−1^·m^−2^ light intensity, with medium change every four weeks. For tuberization, buds at the 2^th^ ~ 4^th^ nodes from the shoot apex from two-week-old ‘E3’ were grown on the P0 medium for two weeks, and then transferred to the potato tuber induction medium (PTI: MS + 6 g·L^−1^ sucrose + 3 g·L^−1^ agar). The culture was maintained for 60 days at 18 ~ 20℃ in 38 μm·sec^−1^·m^−2^ on a 8 h/16 h day/night cycle (short day condition, SD) for tuberization.

### Isolation and qRT-qPCR analyses of *DoBBX2* and *DoBBX8*

All fresh samples were immediately frozen in liquid nitrogen after collection and stored at -80℃ for the expression analysis of *DoBBX2* and *DoBBX8*.

The total RNA was extracted from the leaves at the 3^th^ ~ 4^th^ nodes from the four-week-old *D. opposita* ‘Tiegun’ using the RNA purification kit (TaKaRa, China). *DoBBX2* and *DoBBX8* were amplified using primers designed in Primer Premier 5.0 (Premier, Canada). The full CDS sequences of *DoBBX2* and *DoBBX8* were cloned into PBI121 with the homologous recombination technology (Vazyme, Nanjing, China).

The tissue-specific expression patterns of *DoBBX2* and *DoBBX8* were examined from root (R), stem (S), leaf (L), main bud (MB) and accessory bud (AB) of four-week-old *D. opposita* ‘Tiegun’ as well as the MBs 0, 7, 14, 28, 35, and 42 d after microtuber induction. The 3^rd^ leaf from the shoot apex of the 4-week old *D. opposita* ‘Tiegun’ plants under 16 h light/8 h dark was collected in the dark period every 3 h for the daily expression patterns. Then, the plants were transferred to continuous light for circadian expression analysis. The 3^rd^ leaf was collected every 3 h for 60 h after the initial adaptational period (8 h) for continuous light. The experiments were repeated three times, each time with 10 planets.

cDNAs were synthesized using a HiScriptII 1st Strand cDNA Synthesis Kit (Vazyme, Nanjing, China) and transcript levels were quantified with qPCR run in the Roche Real-Time PCR Detection System with the AceQ qPCR SYBR Green Master Mix (Vazyme, Nanjing, China). All reactions were done in triplicate. The primers for gene isolation and qRT-PCR analysis were listed in Supplementary Table 5.

The SMART (http://smart.embl-heidelberg.de/) was employed to determine conserved domains of DoBBX2 and DoBBX8 proteins. The NJ tree was constructed using MEGA 7.0 software with 1,000 bootstrap replicates among full-length amino acid residues of *BBX2/COL5* and *BBX8/COL8* homologous genes in *D. opposita* ‘Tiegun’ (*Do*), *D. alata* (*Da*), *D. rotundata* (*Dr*), *Phoenix dactylifera* (*Pf*), *Elaeis guineensis* (*Eg*), *Dendrobium officinale* (*Dof*), *Phalaenopsis equestris* (*Peq*), *O. sativa (Os)*, *A. thaliana* (*At*), *S. tuberosum* (*St*), *N. nucifera* (*Nn*), *Vitis vinifera* (*Vv*), *Malus domestica* (*Md*) and *Populus trichocarpa* (*Ptr*) (Supplementary Table 6).

### Transformation in potato and tuberization

Slices of tuber from ‘E3’ potato were used for *Agrobacterium tumefaciens*-mediated transformation. The vector containing *35S::DoBBX2* and *35S::DoBBX8* were transformed into *A. tumefaciens* strain LBA4404 using electroporation, individually. The overexpression (OE) lines were selected on kanamycin (100 mg·L^−1^) MS medium, verified by semi-RT-PCR and qRT-PCR (specific primers were listed in Supplementary Table 5), and propagated by growing single nodes on P0 medium under LD. Two-week-old seedlings of OE lines and ‘E3’ were transferred to SD or dark for the observation of tuberization, and tuberization rate-related parameters were documented including tuberization time, number of tubers per plants, and tuber weight per tuber. The experiments were repeated three times and each time with 10 plants for each treatment, i.e., SD, or dark.

### Statistical analyses

Statistical analysis of collected data was carried out by Excel and SPSS in this chapter. The Fisher LSD test (*P* < 0.05) were performed to test whether there were significant differences at *P* = 0.05 in MS Excel 2016 (Microsoft, https://www.microsoft.com) and SPSS V22 (ANOVA) (IBM SPSS Statistics version 22, SPSS lnc, Chicago, IL, 2014). Boxplot statistics were computed with “paired comparison plot” of Origin 2023 (Origin software; Microcal Software, Northampton, MA).

## Results

### Identification and characteristics of *BBX* genes in yam

#### Identification and phylogenetic analysis of *BBX* genes in yam

Sequence identity analyses using HMM identified 20, 20, and 19 *BBX* genes in *D. rotundata*, *D. alata*, and *D. opposita*, respectively (Supplementary Tables 1—3).

**Fig. 1:**
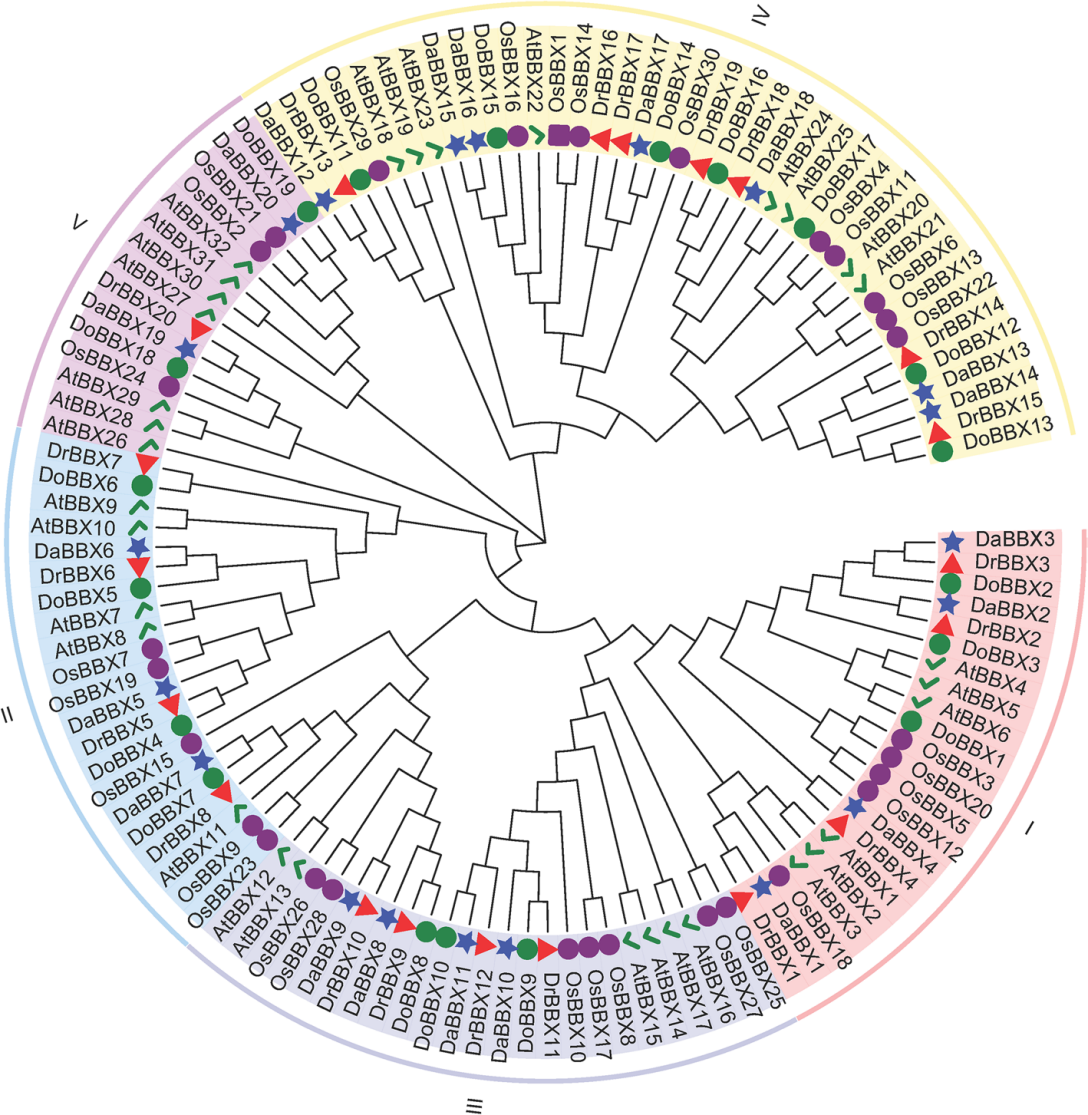
Phylogenetic relationships of BBXs. The full-length amino acid sequences of *BBX* genes in *A. thaliana*, *O. sativa*, *D. rotundata, D. alata*, and the translated amino acid sequences of *BBX* unigenes in *D. opposita* ‘Tiegun’ transcriptome were used for NJ phylogeny reconstruction with 1000 bootstrap replicates. The green check, purple circle, red triangles, blue stars and green circles represent AtBBXs, OsBBXs, DrBBXs DaBBXs and DoBBXs, respectively. Groups I, II, III, IV, and V are shaded in red, blue, yellow, prey, and purple, respectively

The phylogenetic analysis suggested that there are five groups of *the BBX* families in *D. rotundata*, *D. alata*, and *D. opposita* (Fig. 1, Supplementary Tables 1–3). Group IV had the most members (7 Dr, 7 Da and 7 Do), while Group V had the fewest members (1 Dr, 2 Da and 2 Do) (Fig. 1). In addition, the orthologous genes identified among three *Dioscorea* species are highly conserved.

#### Chromosomal location and gene duplication events of BBX in yam

**Fig. 2:**
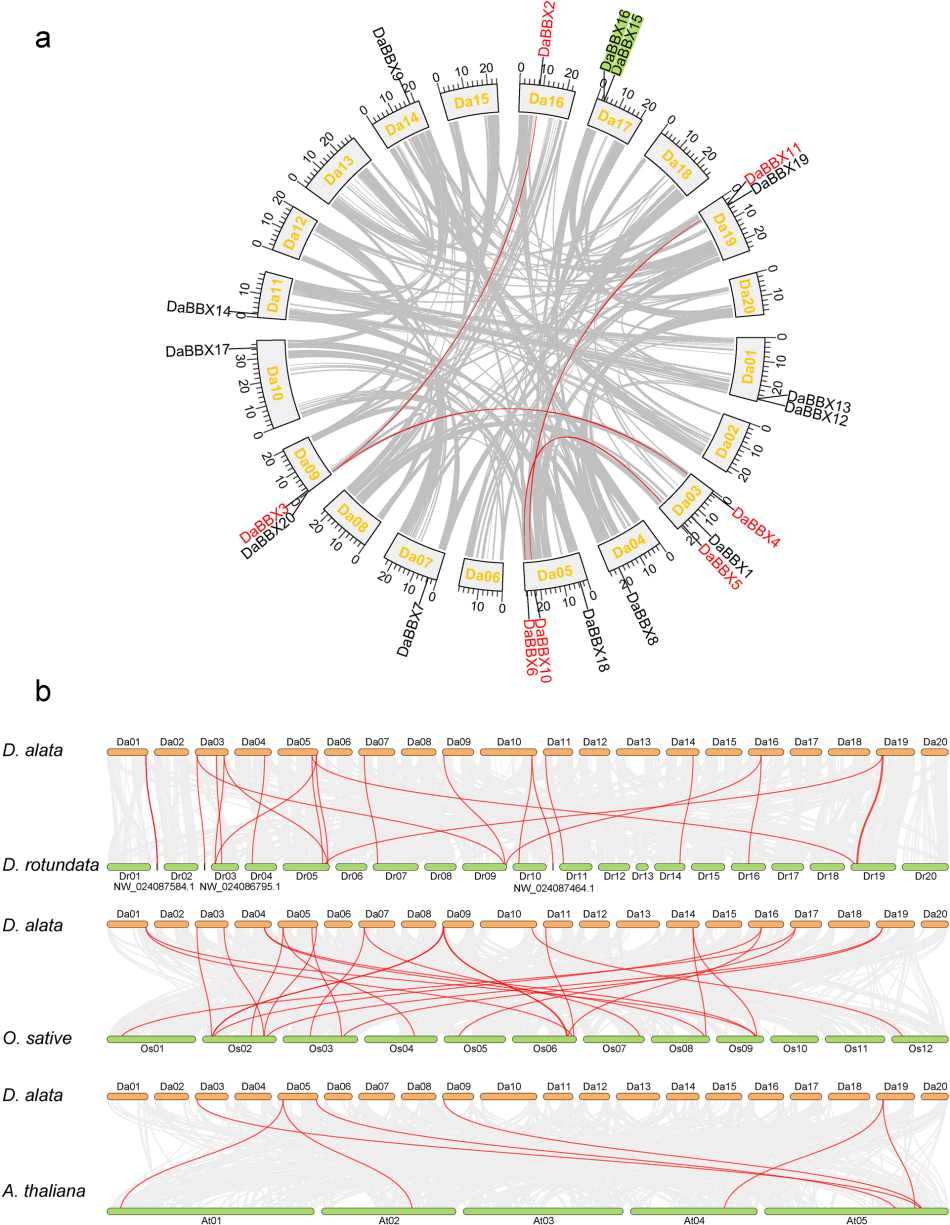
The expansion and evolution analysis of *BBX* genes in the greater yam. The grey lines and gene names indicate duplicated blocks and red lines and names indicate duplicated gene pairs of *BBXs*. For each species, chromosome is indicated by the first letter of species name and chromosome number. **a** Intraspecific colinearity analysis in DaBBXs showing physical positions of 20 *DaBBXs* gene mapped onto the greater yam chromosome. **b** Collinearity between *D. rotunda* and *D. alata*, *D. alata* and rice (*O. sativa*), and *D. alata and A*. *thaliana*

In *D. rotunda*, 17 *BBX* genes are located on 11 of the 20 chromosomes, whereas the other three *DrBBX*s are found on scaffolds. All of the 20 *BBX* genes are all located on 12 of the 20 chromosomes in *D. alata* (Fig. 2a and Supplementary Figure 1). Intraspecific collinearity analysis in *D. alata* suggests that there are both segmental and tandem duplications in this species, producing four and one BBX gene pairs, respectively (Fig. 2a).

Homologous gene analysis between *D. alata* and *D. rotundata* showed that 23 Da-Dr orthologous gene pairs were located in collinear regions (Fig. 2b, Supplementary Table 4). While 23 homologous genes pairs are colinear, genes are slightly different in their locations on the chromosomes between *D. alata* and *D. rotundata*.

*D. alata* has more colinear *BBX* genes with rice (26 pairs) than with *A.thaliana* (7 pairs). The *DaBBX* genes showed one-to-one (6), two-to-one (17), and three-to-one (3) corresponding relationships with *BBX* genes in rice, while showed one-to-one (1), two-to-one (2), and one-to-two (4) corresponding relationships with *BBX* genes in *A. thaliana*. The results indicated that loss and expansion of *BBX* genes were discovered in *D. alata* when compared with *O. sativa* and *A. thaliana*.

Subsequently, the *Ka*, *Ks*, and *Ka/Ks* of the 34 homologous gene pairs in *D. alata* and *D. rotundata* were calculated to evaluate their molecular evolutionary rates (Supplementary Table 4). Previous studies have shown that yam possessed a genome-wide duplication event (the value of *Ks* is approximately 1.21) [25, 51]. The *Ks* values of *DaBBX5*-*DrBBX6*, *DaBBX6*-*DrBBX5*, *DaBBX10*-*DrBBX12*, *DaBBX11*-*DrBBX11*, *DaBBX5*-*DaBBX6*, *DaBBX10*-*DaBBX11*, *DrBBX5*-*DrBBX6*, and *DrBBX11*-*DrBBX12* were 1.6581, 2.1292, 2.3224, 2.5170, 1.8292, 2.0216, 1.994, and 2.7183, respectively. This indicated that these gene pairs were derived from genome-wide duplication events shared by *D. alata* and *D. rotundata*. The *Ks* values for the other paralogs, including *DaBBX15*-*DaBBX16* and *DrBBX18*-*DrBBX19*, were 0.0190 and 0.0062, respectively, suggesting that they were derived from the ancient duplication events. The *Ka/Ks* values of all *DrBBX/DaBBX* gene pairs were less than 1.0 (Supplementary Table 4), indicating that they were under strong purifying selection during their evolution and a conserved evolutionary pattern was shared among *BBX* gene family in yam.

#### Conservative domain, motif and gene structure analyses of DrBBXs and DaBBXs

**Fig. 3:**
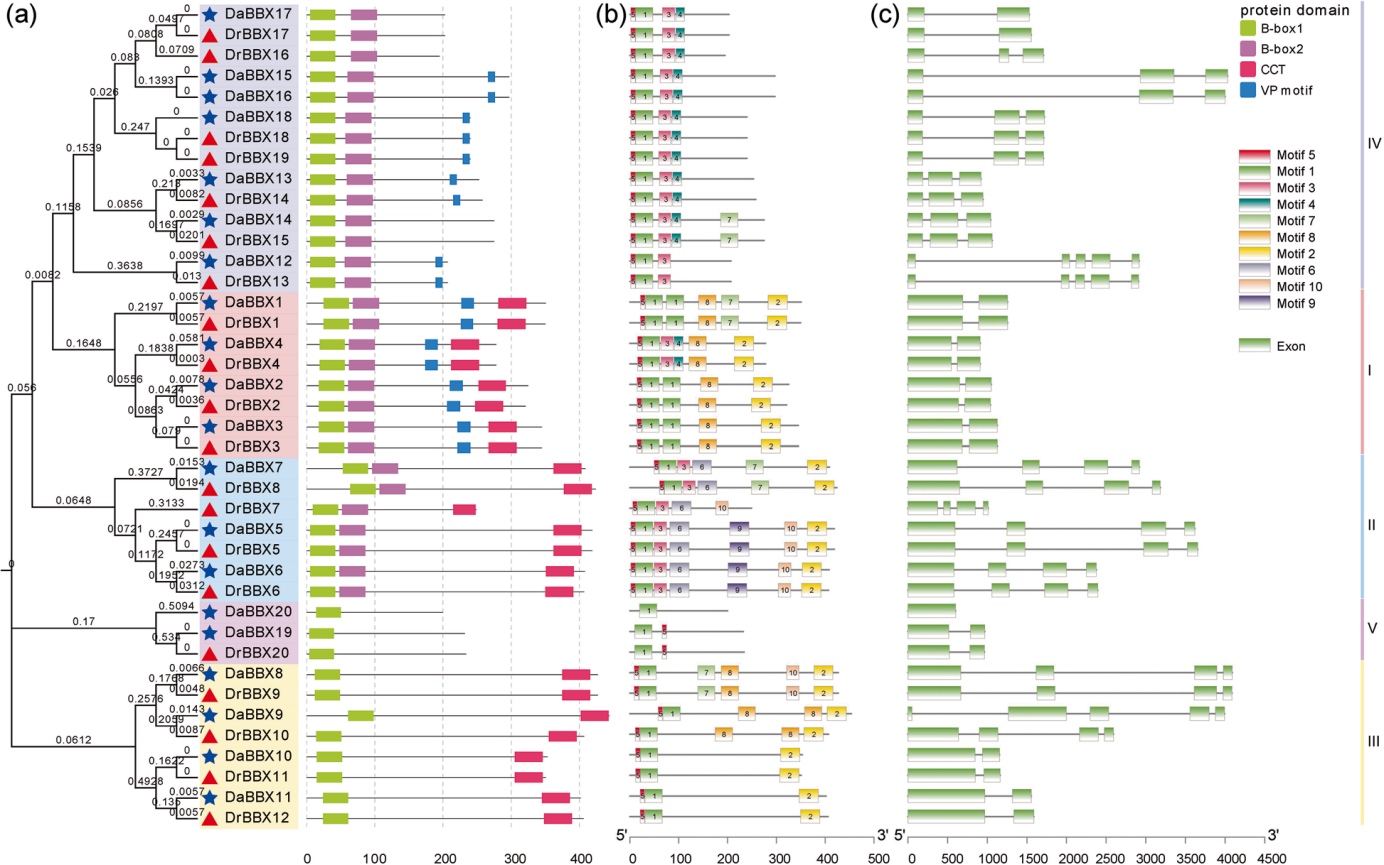
Protein domain diagram, motif phases and gene structure of *DrBBX* and *DaBBX genes* mapped on phylogenetic trees. **a** The conserved domain distribution of *DrBBXs* and *DaBBXs*. The groups I, II, III, IV, and V are shaded in red, blue, yellow, grey, and purple, respectively. The red triangles and blue stars represent DaBBXs, and DaBBXs, respectively, and branch lengths are shown close to the branch nodes. **b** The conserved motifs on DrBBX and DaBBX proteins. **c** The exon and intron phases of *DrBBX* and *DaBBX* genes. The legend is shown in the upper right corner

In the predicted protein domains, there is conserved B-box1 domain in all of DrBBX and DaBBX proteins, in the *BBX* gene groups I, II, III, IV and V, however, the conserved domains included the B-box1 + B-box2 + CCT, B-box1 + B-box2 + CCT, B-box1 + CCT, B-box1 + B-box2, and B-box1 domain combinations, respectively. Besides, all members in group I and 9 individual members in Group IV contained a valine-proline (VP) motif in their C-terminal region (Fig. 3a, Supplementary Figure 2). Protein sequence alignment and logo analysis show that B-box1, B-box2, CCT domains and VP motifs are highly conserved (Supplementary Figures 2 and 3).

*DrBBXs* and *DaBBXs* from same groups are similar in motif number, exon/intron number, length and arrangement (Fig. 3b,c). Motif 1 + Motif 10 are present in all of the DrBBX and DaBBX groups, Motif 2 is present in group I, II and III, Motif 3 + Motif 4 are present in group I, II and IV, whereas Motif 8 was specific in group I and Motif 6 + Motif 9 was specific in group II. Motif 5 was widely distributed in all of the DrBBX and DaBBX members (except DaBBX20), while Motif 7 was only distributed in DaBBX1, DaBBX7, DaBBX8, DaBBX14, DrBBX1, DrBBX8, DrBBX9, and DrBBX15 (Fig. 3b,c).

#### *Cis*-elements in the promoter regions of *DrBBXs* and *DaBBXs*

**Fig. 4:**
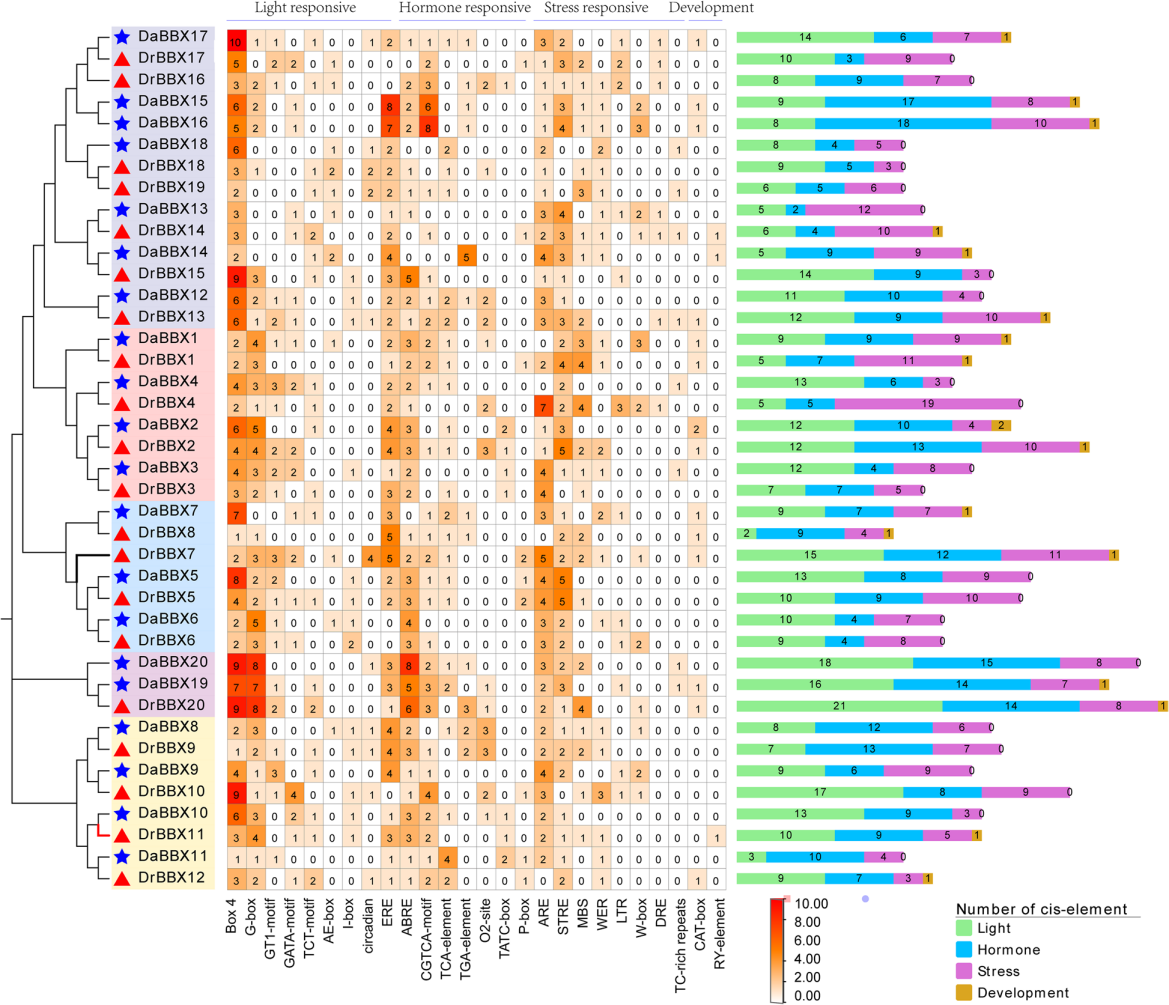
*Cis*-elements of *BBX* genes in *D. rotundata* and *D. alata* mapped on the phylogenetic tree of DrBBXs and DaBBXs, as in Fig. 3. The middle panel is the heat map showing the levels of *cis*-acting element enrichment. The chart on the right is a proportional map showing the abundance of *cis*-elements: the green rectangle, light responsive *cis*-elements; the blue rectangle, hormone responsive *cis*-elements; the orchid rectangle, stress responsive *cis*-elements, and the orange rectangle, growth and development related *cis*-elements

We identified 28 *cis*-elements. Besides the conventional *cis*-acting elements (TATA-box, CAAT-box) in the promoter, the other 26 *cis*-acting elements include 8 light responsive, 8 hormone-responsive, 8 stress responsive, and 2 growth and development groups (Fig. 4). The most frequent elements were Box 4 (the total number was 176) and G-Box (95) (involved in light responsiveness), ABRE (ABA responsive element, 98), and ARE (essential for the anaerobic induction, 92).

### Excavation and functional analysis of *BBXs* in microtuber formation in *D. opposita* ‘Tiegun’

#### Excavation of differentially expressed *BBXs* during the microtuber formation

**Fig. 5:**
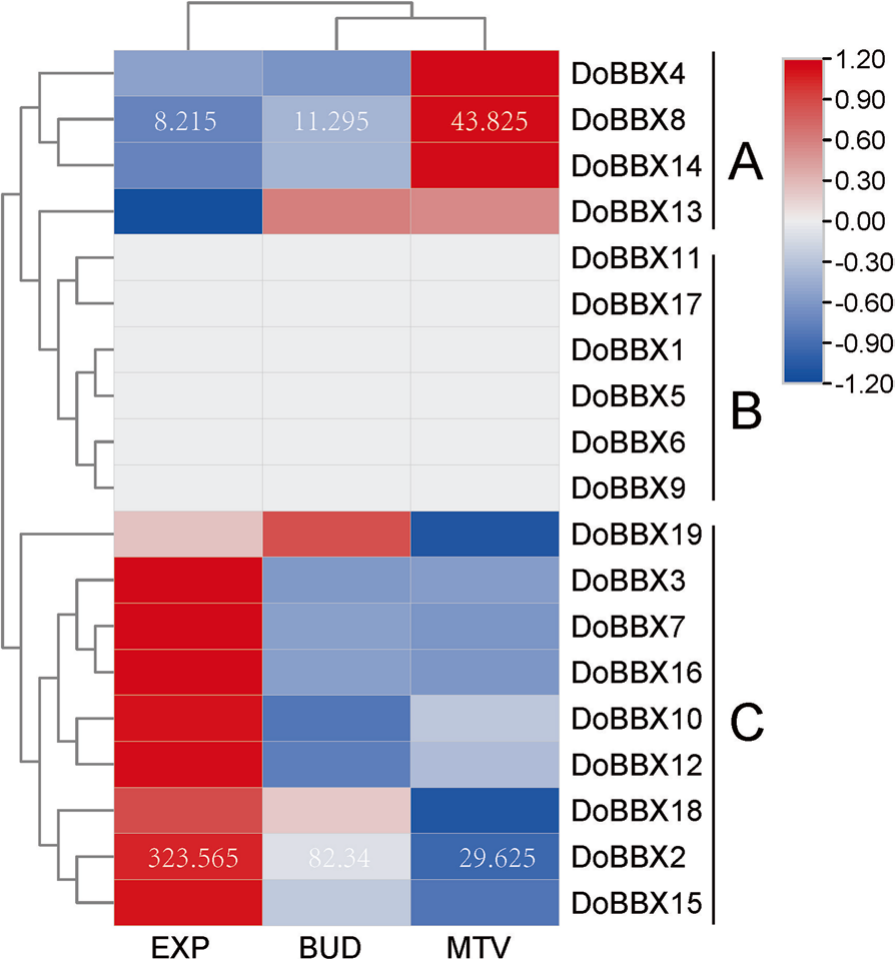
Expression heatmap of *DoBBX*s genes during the microtuber formation in *D. opposita* ‘Tiegun’ of different developmental stages. EXP: initial explants (0 d), BUD: axillary bud proliferation after three weeks (21 d), and MTV: microtuber visible after four weeks (28 d)

Based on the data obtained from RNA-seq during the microtuber formation in *D. opposita* ‘Tiegun’, the accumulation of *DoBBX*s during microtuber formation were depicted by heat map (Fig. 5). On the basis of the expression patterns of the *BBX* genes during the microtuber formation, the *DoBBX*s were clustered into three main classes: class A, class B and class C (Fig. 5). Six *DoBBX* members in class B were nearly absent during microtuber formation. Whereas 13 *DoBBX* genes showed differentially expression patterns during microtuber formation, which included Class A (*DoBBX*8, *DoBBX*14 and *DoBBX*4) were upregulated, and Class C (*DoBBX2*, *DoBBX3*, *DoBBX7*, *DoBBX10*, *DoBBX12*, *DoBBX15*, *DoBBX16*, and *DoBBX18*) were down regulated. Among them, *DoBBX2* transcripts were the highest at EXP stage, and a significant reduction (~ 10.922-fold) was found from EXP to MTV stages. Besides, we observed the highest increase of *DoBBX8* expression (~ 3.880-fold) from EXP to MTV stages. Further study would be focused on *DoBBX2 and DoBBX8*.

#### Spatial expression patterns of *DoBBX2* and *DoBBX8*

*DoBBX2* and *DoBBX8* were isolated from *D. opposita* ‘Tiegun’ and submitted to NCBI’s GenBank (*DoBBX2/DoCOL5*, NCBI Accession No: CP897490; *DoBBX8/DoCOL8*, NCBI Accession No: CP897491). The full-length sequence of *DoBBX2* cDNA was 1332 base pair (bp), ORF was 1041 bp length and encoded a deduced protein of 347 amino acids (aa) residues, with two B-box domains at the N-terminus and one CCT domain at the C-terminus. The full-length cDNA sequence of *DoBBX8* was 1811 bp with a 1281 bp of ORF, and the deduced amino acids were 427 aa with one B-box domains at the N-terminus and one CCT domain at the C-terminus (Supplementary Figure 4).

**Fig. 6:**
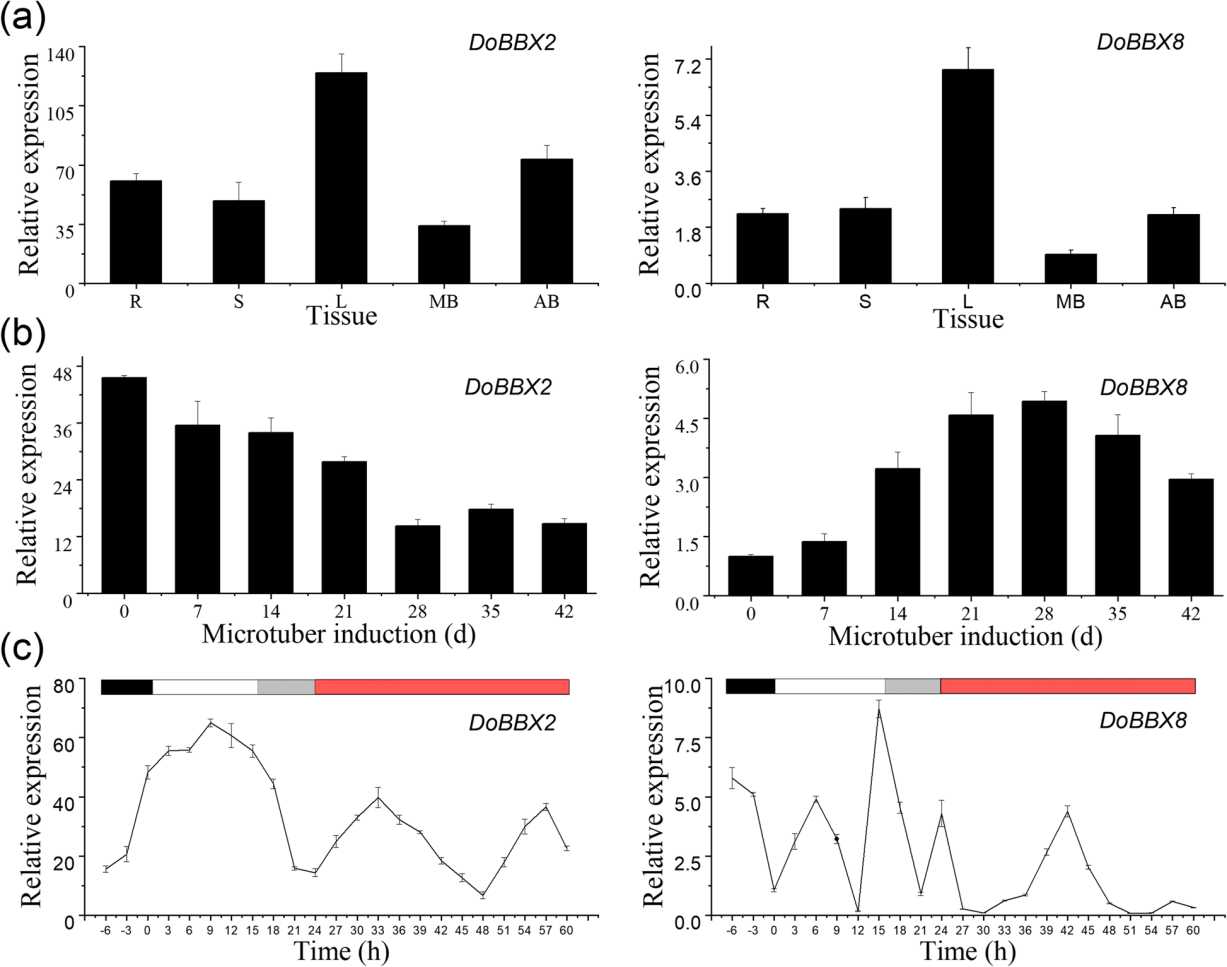
Expression patterns of *DoBBX2* and *DoBBX8* gene. **a** *DoBBX2* and *DoBBX8* mRNA levels relative to *DoActin* in root (R), stem (S), leaf (L), main bud (MB) and accessory bud (AB). **b** The expression patterns of *DoBBX2* and *DoBBX8* gene during the microtuber formation under different light conditions: Black box: dark. White box: light. Grey box: 8-h light adaptations. Red box: continuous light phase. **c** The diurnal and circadian regulation of *DoBBX2* and *DoBBX8* gene expression under a 14-h photoperiod and then continuous light. Error bars = SEM

Similar expression patterns were observed for the two *DoBBX* genes in 5 tissues with the highest expression in leaf (L), moderate in root (R), stem (S) and accessory buds (AB), and the lowest in main buds (MB) (Supplementary Figure 5, Fig. 6a). Specifically, the expression levels of *DoBBX2 and DoBBX8* in L was ~ 3.90 times and ~ 7.61 times that in MB, respectively. The transcript levels of *DoBBX2* were significantly higher than that of *DoBBX8*. This finding implied *DoBBX2 and DoBBX8* may participate in the regulation of microtuber formation by receiving signals in leaf and then travel to the tissues forming microtubers.

In order to verify the involvement of *DoBBX2 and DoBBX8* genes in microtuber formation, the expression levels of these two *DoBBX*s were detected at five stages during microtuber formation (Fig. 6b). The *DoBBX2* expression gradually decreased. However, the expression level of the *DoBBX8* increased from EXP stage (0 d) to MTV stage (28 d), then decreased from early to late expansive stages (35d to 42 d). It showed that *DoBBX2 and DoBBX8* were involved in microtuber formation through different patterns in *D. opposita* ‘Tiegun’.

Given that numerous plant BBX proteins are implicated in multiple light-regulated growth and developmental processes, we then sought to study the daily oscillation and circadian expression pattern of *DoBBX2 and DoBBX8* (Fig. 6c). The changes in the expression profiles of *DoBBX2 and DoBBX8* were observed in the day/night cycle oscillate following about 24-h rhythm. The transcript level of *DoBBX2* increased during the day and reached a maximum in the middle of the light phase, whereas that of *DoBBX8* had two main peaks throughout the 24-h diurnal cycle, the first with maximal expression in the light phase and the second with maximal expression in the end of light phase (the point of 15 h under light) or in the beginning of dark phase (before -6 h under dark, not shown). In the 8 h light adaptations period, the transcript level of *DoBBX2* decreased, while that of *DoBBX8* decreased first and then increased. During the continuous 44 h light period, the expression profiles of *DoBBX2* were in synchrony with those observed during the diurnal cycle, but the maximum values were decreased. The expression of *DoBBX8* was altered by prolonging the total duration at the phase of diurnal cyclic. It suggested that the diurnal expression of *DoBBX2* was not directly regulated by light, but by the circadian clock; while diurnal oscillation of *DoBBX8* expression required a dark period.

#### Overexpression of *DoBBX2* and *DoBBX8* regulates tuberization through photoperiodic pathway in potato

We produced more than 10 transgenic potato lines with *35S::DoBBX2* and *35S:: DoBBX8* constructs as detected using semi-PCR and qRT-PCR (Supplementary Figure 6).

**Fig. 7:**
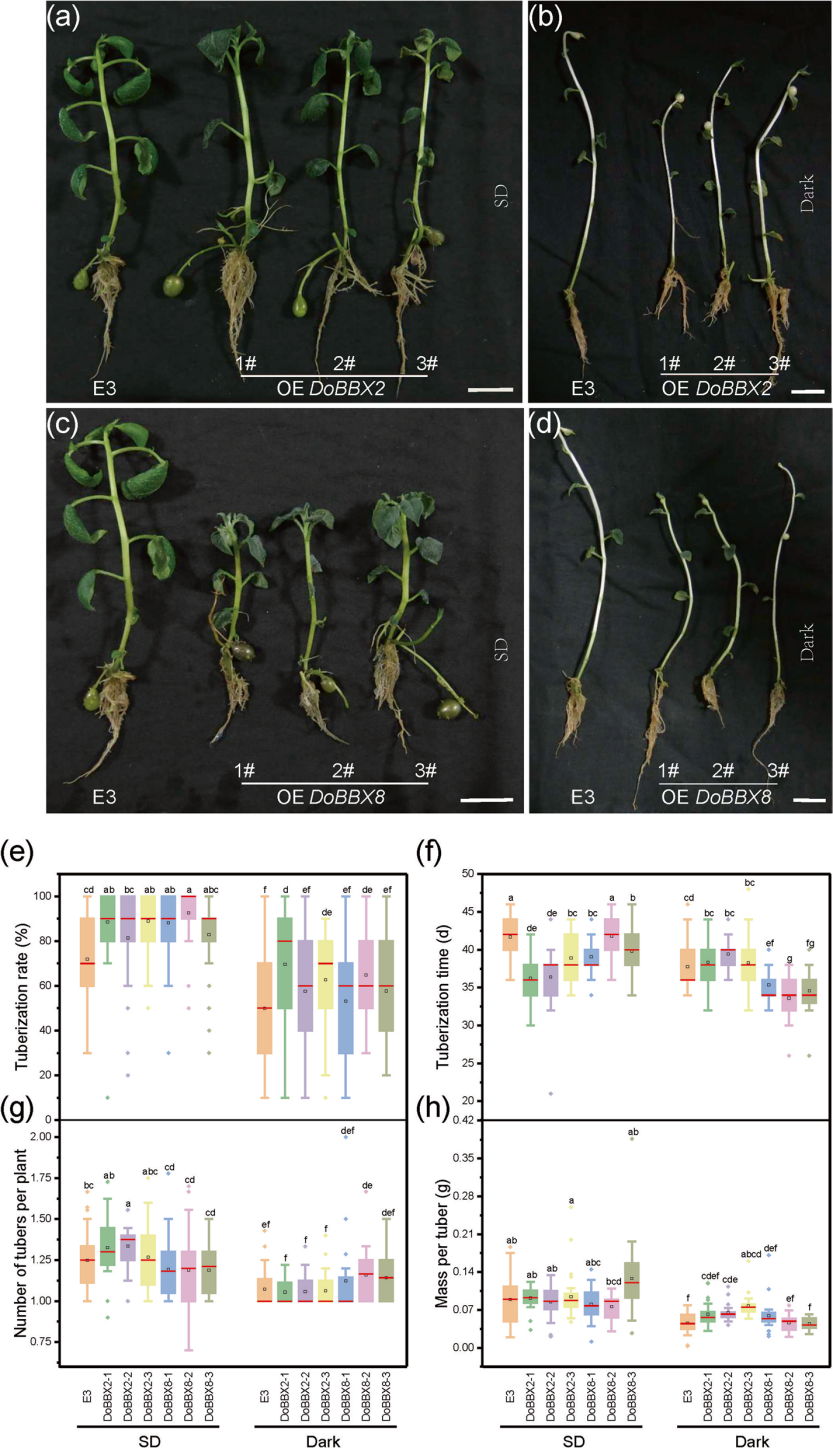
Tuberization in the control (‘E3’) and transgenic potato plants expressing *DoBBX2* and DoBBX8 transgenes (OE *DoBBX2 and* OE *DoBBX8*) under the SD and dark conditions. **a**-**d** The seedlings and potato tubers of ‘E3’, *DoBBX2 and DoBBX8* overexpression lines (*DoBBX2-1*, *DoBBX2-*2*, DoBBX2-*3, *DoBBX8-1*, *DoBBX8-*2 and *DoBBX8-*3) after 60 days of growth under the SD and Dark conditions, bar = 2 cm. **e**, **g**, **h** Boxplots of tuberization rate, the number of tubers per plant and the mass per tuber after 60 days of growth under the SD and Dark conditions. **f** The boxplots of tuberization time under the SD and Dark. The red line within the boxplot marks the median (50th percentile) while the black box within the box marks the mean

The tuberization in transgenic and control potato lines were investigated under both SD and dark conditions (Fig. 7). Almost all of the transgenic lines (except for *DoBBX8-2*) formed tuber earlier than 'E3' under SD (Fig. 7a,c,f). The OE lines of *DoBBX2-1*, *DoBBX2-2*, *DoBBX2-3*, *DoBBX8-1* and *DoBBX8-3* formed tubers after 21, 34, 34, 34, 34 days of being transferred to SD, respectively, while a minimum of 36 days was required for ‘E3’. Under SD for 60 days, we observed a higher percentage of tuberization in the OE lines of *DoBBX2* and *DoBBX8* than in ‘E3’ (Fig. 7e). In addition, tuber yield was greater in the OE lines of *DoBBX2-1*, *DoBBX2-2* and *DoBBX2-3* than in the ‘E3’, with higher average of tubers produced per plant but without changing weight per tuber (Supplementary Figure 7, and Fig. 7g,h). However, the OE of *DoBBX8* produced fewer tubers per plant (Fig. 7g,h). It indicated that both *DoBBX2* and *DoBBX8* advanced tuberization under SD, while number of tubers per plant was higher in OE *DoBBX2* lines compared to ‘E3’, but it was slightly reduced in OE *DoBBX8* lines.

Under dark, the tuberization time of ‘E3’ and OE *DoBBX8* lines was significantly earlier than that under SD (Supplementary Figure 7, Fig. 7b,d,f). Under dark for 60 days, the tuberization rate, mass per tuber and yield were all reduced in ‘E3’ and OE lines (Fig. 7e,g,h). The number of tubers per plant in OE *DoBBX8-2 and DoBBX8-3* lines had no significant change, while it demonstrated an obvious reduction in the ‘E3’ and OE *DoBBX2* lines than that under SD (Fig. 7g). This indicates that although both over-expression of *DoBBX2* and *DoBBX8* can accelerate tuber formation under dark, only the overexpression of *DoBBX8* enhanced the promoting effect under dark on tuber induction.

## Discussion

The BBXs are zinc-finger transcription factors and play vital roles in plant growth, development and response to biotic and abiotic stresses [52–54]. *BBX* gene family has been identified from several plant species, such as *Arabidopsis* [18], rice [55], orchid [56], bamboo [57], tomato [58], potato [59], pepper [60], grapes [61], cottons [62], pear [63], and apple [64]. However, little information is known about the *BBX* gene family in yam, which is one of the important tuberous crops. In this study, we performed systematic genome-wide identification and analyses of the *BBX* gene family in three yam species. Based on the transcriptome data during microtuber formation in *D. opposita* ‘Tiegun’, we selected two candidate genes for further investigation. Furthermore, we focus on the expression patterns and the potential function of the two candidate genes in potato tuberization.

In the present species, segmental duplication counts for 20% (4 Dr-Dr paralogs) of *DrBBX* and 25% (5 Da-Da paralogs) of *DaBBX* (Supplementary Table 4 and Fig. 2). The percentages are smaller than those in rice (60%, 18 *OsBBX*) [55], *Phyllostachys edulis* (88.89%, 24 *PeBBX*) [57], *Solanum lycopersicum* (40%, 12 *SlBBXs*) [58], but greater than those in *D. officinale* (10.53%, 2 *DofBBXs*) and *P. equestris* (12.50%, 2 *PeqBBXs*) [56]. It may be the reason for the difference in *BBXs* number among these species.

We found five groups of the *BBX* genes and they varied in conserved domains, motifs and gene structures. Group IV with the largest number of the *BBX* genes in *D. rotundata*, *D. alata*, *Arabidopsis* and rice, it may have undergone a much greater gene expansion [17]. However, the reasons are unclear. The differences between groups might be linked with a wide functional diversity in *BBX* gene family [65].

The different *cis*-regulatory elements in the promoter regions may also be important for functional diversity [66]. The G-box and its variants in the promoter region of *BBX* gene are the binding sites of central regulators like HY5 and PIFs of photomorphogenesis [67]. The homologous genes of *DoBBX2* and *DoBBX8* in *D. rotundata* and *D. alata* possess the G-box as well as the ABA/GA-responsive *cis*-elements. 85% of *DaBBXs* (17) and 90% *DrBBXs* (18), including homologous genes of *DoBBX2* and *DoBBX8*, have ABRE motifs, whereas 85% of *DaBBXs* (17) and 85% of *DrBBXs* (17) contain CGTCA-motif (Fig. 4). The *BBX1/CO* gene was initially identified in *Arabidopsis* as an important regulator of flowering in the photoperiodic pathway [19, 68, 69] and also play a role in regulating the tuber formation in potato [16, 53, 55]. Therefore, *DoBBX2* and *DoBBX8* may have the potential functions of photoperiodic and hormone-regulated tuberization.

Our spatio-temporal expression analyses indicate that DoBBX2 and DoBBX8 proteins may function as components of circadian clock signals during microtuber formation. This is consistent with studies in *Arabidopsis* and rice [59, 70]. However, specific functions regulated by the circadian clock in *DoBBX2* and *DoBBX8* have not yet been clarified.

The length of the dark period is critical for tuberization [15]. *D. opposita* ‘Tiegun’ MBs were previously induced to form microtuber under dark [8]. Tubers of potato plants overexpressing *DoBBX8* (the OE *DoBBX8* lines) form earlier under dark than under the SD (Fig. 7). Surprisingly, the early tuber formation in the OE *DoBBX8* lines occurs in both SD and dark conditions. The overexpression of *DoBBX8* also increases the number of tubers per potato plant under dark conditions. However, *DoBBX2* is down regulated during the microtuber formation, accelerating tuberization in potato under SD, but delaying tuberization under dark. Besides, *DoBBX2* increases the tuber yield per plant both under SD and dark (Supplementary Figure 7). Our results are similar to those of the overexpression of *NnCOL5* in potato, which affects the expression levels of *NnCOL8*, and the related genes in CO-FT and GA signal pathways [22]. Therefore, one of the future studies may focus on elucidating the regulatory relationships between *DoBBX2*, *DoBBX8*, hormone-regulated and photoperiod related genes.

## Conclusion

In this study, we conducted a systematic genome-wide analysis of the *BBX* gene family in three yam species and identified 20 *DrBBX*, 20 *DaBBX* and 19 *DoBBX* genes in *D. rotundata*, *D. alata* and *D. opposita* ‘Tiegun’ transcriptome, respectively. The *BBX* genes form five major groups that are characterized by duplications, conserved domains, motifs, gene structure and *cis*-elements. Our RNA-*seq* data suggest that *DoBBX2* and *DoBBX8* are functional *BBX* genes during *D. opposita* ‘Tiegun’ microtuber formation under SD and dark condition. The overexpression of *DoBBX2* and *DoBBX8* in potato accelerates tuberization under SD and increase the number of tubers per plant under both SD and dark. Overall, this study forms the basis for future functional characterizations of yam *BBX* gene family, especially regarding regulation of tuberization *via* the photoperiodic pathway.

## Supplementary Information

**Additional file 1: Supplementary Table 1.** The information of *BBX* family genes in *D. rotundata*. **Supplementary Table 2.** The information of *BBX* family genes in *D. alata*. **Supplementary Table 3.** The information of *BBX* family genes in *D. opposita* ‘Tiegun’. **Supplementary Table 4.** Homologous *BBX* gene pairs between *D. alata, D. rotundata, O. sativa *and* A. thaliana*. The homologous gene pairs were identified by the results of BLAST and collinearity analysis. When both *Ka* and *Ks* were equal to 0, *Ka/Ks* was considered equal to 1. When only *Ks* was equal to 0, it was marked as *Ka >> Ks*. Segmental means that the gene might arise from segmental duplication. Tandem means that the homologous sequences in close genomic proximity. **Supplementary Table 5.** Primers used for gene cloning, semiqRT-PCR and qRT-PCR detection of *DoBBX2* and *DoBBX8*. The double underscores represent the pBI121 plasmid based linker sequences. The restriction endonuclease recognition sites for *Sac* I and *Xba* I were included in the respective oligonucleotide primers, which are markered with single underlines. **Supplementary Table 6.** Accession numbers of the protein sequences used in phylogenetic analysis of DoBBX2 and DoBBX8. *Do: D. opposita* 'Tiegun', *Da*: the greater yam (*D. alata*), *Dr*: the white Guinea yam (*D. rotundata*), *Pd: P. dactylifera, Eg: E. guineensis, Dof: D. officinale, Peq: P. equestris, Os: O. sativa, At: A. thaliana, St: S. tuberosum, Nn: N. nucifera, Vv: V. vinifera, Md: M. domestica, Ptr: P. trichocarpa*.

**Additional file 2: Supplementary Figure 1.** Chromosomal distribution of *DrBBXs* and *DaBBXs*. Chromosomal mapping was based on the physical position in the white *D. rotundata* (Dr) and *D. alata* (Da) chromosomes. The chromosome numbers are presented above each vertical bar. The scale on the left is in base pairs (Mb).** Supplementary Figure 2.** Multi-sequence alignment of BBX proteins in *D. rotundata* and *D. alata*. B-box domains are marked by green boxes, CCT conserved domain is markered by an orchid box, VP motifs are marked by blue boxes.** Supplementary Figure 3. **Conserved domains analyses of DrBBX and DaBBX proteins were aligned by a WebLogo program using default parameters. The B-box1, B-box2, and CCT conserved domain logos were obtained by aligning all the 40 BBXs sequences, 29 group I, II, IV sequences, and 23 group I, II, III sequences form the white Guinea yam and the greater yam, respectively. The group I’s VP motif logo was obtained by aligning the sequences of four group I DrBBXs and four group I DaBBXs. The group IV’s VP motif logo was obtained from DrBBX13, DrBBX14, DrBBX18, DrBBX19, DaBBX12, DaBBX13, DaBBX15, DaBBX15, and DaBBX16.** Supplementary Figure 4.** Cloning, domain diagram and phylogenetic analysis of *DoBBX2* and *DoBBX8*. (a) The complete cDNA sequences and amino acid sequences of *DoBBX2* and *DoBBX8*. (b) DoBBX2 and DoBBX8 protein domains. (c) Phylogenetic analysis of *DoBBX2* and *DoBBX8.* *Do*: *D. opposita* ‘Tiegun’, *Da*: the greater yam (*D. alata*), *Dr*: the white Guinea yam (*D. rotundata*), *Pd*: *Phoenix dactylifera*, *Eg*: *Elaeis guineensis*, *Dof*: *Dendrobium officinale*, *Peq*: *Phalaenopsis equestris*, *Os*: *O. sativa*, *At*: *A. thaliana*, *St*: *Solanum tuberosum*, *Nn*: *Nelumbo nucifera*, *Vv*: *Vitis vinifera*, *Md*: *Malus domestica*, *Ptr*: *Populus trichocarpa*. The red diamond represents DoBBX2/DoCOL5 protein in ‘Tiegun’, and the blue triangle represents DoBBX8/DoCOL8 protein in ‘Tiegun’.** Supplementary Figure 5. **Morphological structure of 45 d-old *D. opposita* ‘Tiegun’ plant. (Bar=1 cm). **Supplementary Figure 6. **The transcript levels of *DoBBX2* and *DoBBX8* in 15-days-old control pants and overexpression plants. (a) The quantitative qRT-PCR results. (b) The semi quantitative PCR results. Left panel shows the transcript levels of *DoBBX2* gene in 15-days-old control pants (‘E3’) and *DoBBX2 *overexpression plants (*DoBBX2*-3, *DoBBX2*-1 and *DoBBX2*-2). The grouping of gels/blots cropped from different parts of the same gel. Right panel shows the transcript levels of *DoBBX8* gene in 15-days-old control pants (‘E3’) and *DoBBX8 *overexpression plants (*DoBBX8*-2, *DoBBX8*-3 and *DoBBX8*-1). The grouping of gels/blots cropped from different parts of the same gel. The potato *ELONGATION FACTOR 1 α* (*EF1-α*) gene was used as reference gene for primary data normalization. Error bars indicate standard deviations (SD) from three biological replicates. **Supplementary Figure 7. **Tuber yield per plant in controls and the lines overexpressing* DoBBX2* and *DoBBX8*. Data are from three independent biological experiments (*n* = 3 independent biological experiments × 9 growth chambers × 10 individual plants). The red line within the boxplot marks the median (50th percentile) while the black box within the box marks the mean. Significant differences between two means are indicated by Fisher LSD test (*P*<0.05).

**Additional file 3: Supplementary Info File 1.** The semi quantitative PCR result of OE *DoBBX2* and *DoBBX8* lines.

**Additional file 4: Supplementary Info File 2.** The raw data of potato tuberization index.

## Data Availability

The data included in this article and the additional files are available.
