# Supplementary material for: Identification of BBX gene family and its function in the regulation of microtuber formation in yam
Source: BMC Genomics. 2023 Jun 26;24:354. doi: 10.1186/s12864-023-09406-1 (PMC10291778; doi:10.1186/s12864-023-09406-1)
Supplement: Supplementary file 2 — Additional file 2: Supplementary Figure 1. Chromosomal distribution of DrBBXs and DaBBXs. Chromosomal mapping was based on the physical position in the white D. rotundata (Dr) and D. alata (Da) chromosomes. The chromosome numbers are presented above each vertical bar. The scale on the left is in base pairs (Mb). Supplementary Figure 2. Multi-sequence alignment of BBX proteins in D. rotundata and D. alata. B-box domains are marked by green boxes, CCT conserved domain is markered by an orchid box, VP motifs are marked by blue boxes. Supplementary Figure 3. Conserved domains analyses of DrBBX and DaBBX proteins were aligned by a WebLogo program using default parameters. The B-box1, B-box2, and CCT conserved domain logos were obtained by aligning all the 40 BBXs sequences, 29 group I, II, IV sequences, and 23 group I, II, III sequences form the white Guinea yam and the greater yam, respectively. The group I’s VP motif logo was obtained by aligning the sequences of four group I DrBBXs and four group I DaBBXs. The group IV’s VP motif logo was obtained from DrBBX13, DrBBX14, DrBBX18, DrBBX19, DaBBX12, DaBBX13, DaBBX15, DaBBX15, and DaBBX16. Supplementary Figure 4. Cloning, domain diagram and phylogenetic analysis of DoBBX2 and DoBBX8. (a) The complete cDNA sequences and amino acid sequences of DoBBX2 and DoBBX8. (b) DoBBX2 and DoBBX8 protein domains. (c) Phylogenetic analysis of DoBBX2 and DoBBX8. Do: D. opposita ‘Tiegun’, Da: the greater yam (D. alata), Dr: the white Guinea yam (D. rotundata), Pd: Phoenix dactylifera, Eg: Elaeis guineensis, Dof: Dendrobium officinale, Peq: Phalaenopsis equestris, Os: O. sativa, At: A. thaliana, St: Solanum tuberosum, Nn: Nelumbo nucifera, Vv: Vitis vinifera, Md: Malus domestica, Ptr: Populus trichocarpa. The red diamond represents DoBBX2/DoCOL5 protein in ‘Tiegun’, and the blue triangle represents DoBBX8/DoCOL8 protein in ‘Tiegun’. Supplementary Figure 5. Morphological structure of 45 d-old D. opposita ‘Tiegun’ plant. (Bar=1 cm). Suppleme [file 12864_2023_9406_MOESM2_ESM.docx]

**
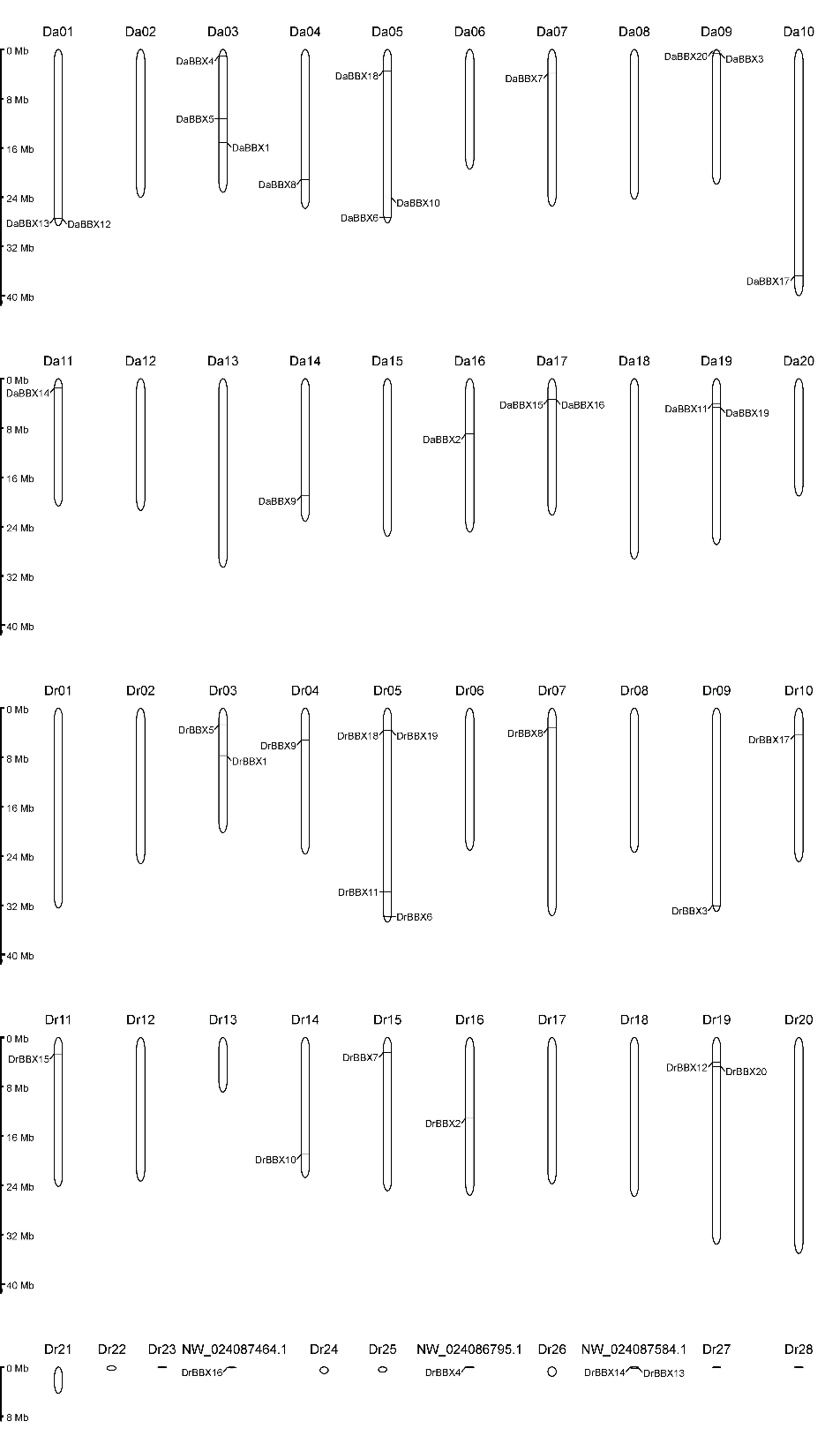
**

**Supplementary Figure 1 Chromosomal distribution of *DrBBXs* and *DaBBXs*.** Chromosomal mapping was based on the physical position in the white *D. rotundata* (Dr) and *D. alata* (Da) chromosomes. The chromosome numbers are presented above each vertical bar. The scale on the left is in base pairs (Mb).


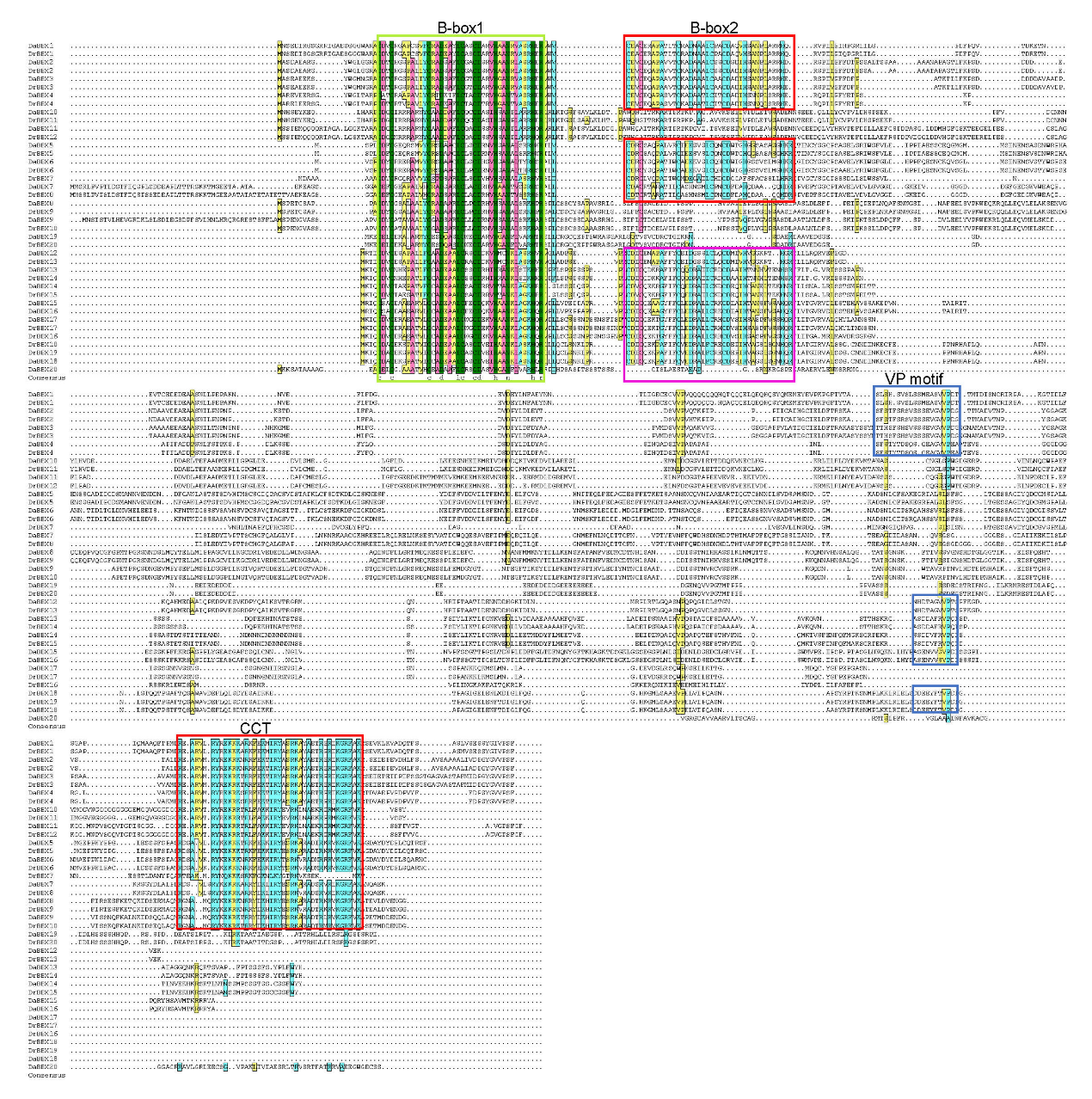


**Supplementary Figure 2 Multi-sequence alignment of BBX proteins in *D. rotundata* and *D. alata*.** B-box domains are marked by green boxes, CCT conserved domain is markered by an orchid box, VP motifs are marked by blue boxes.


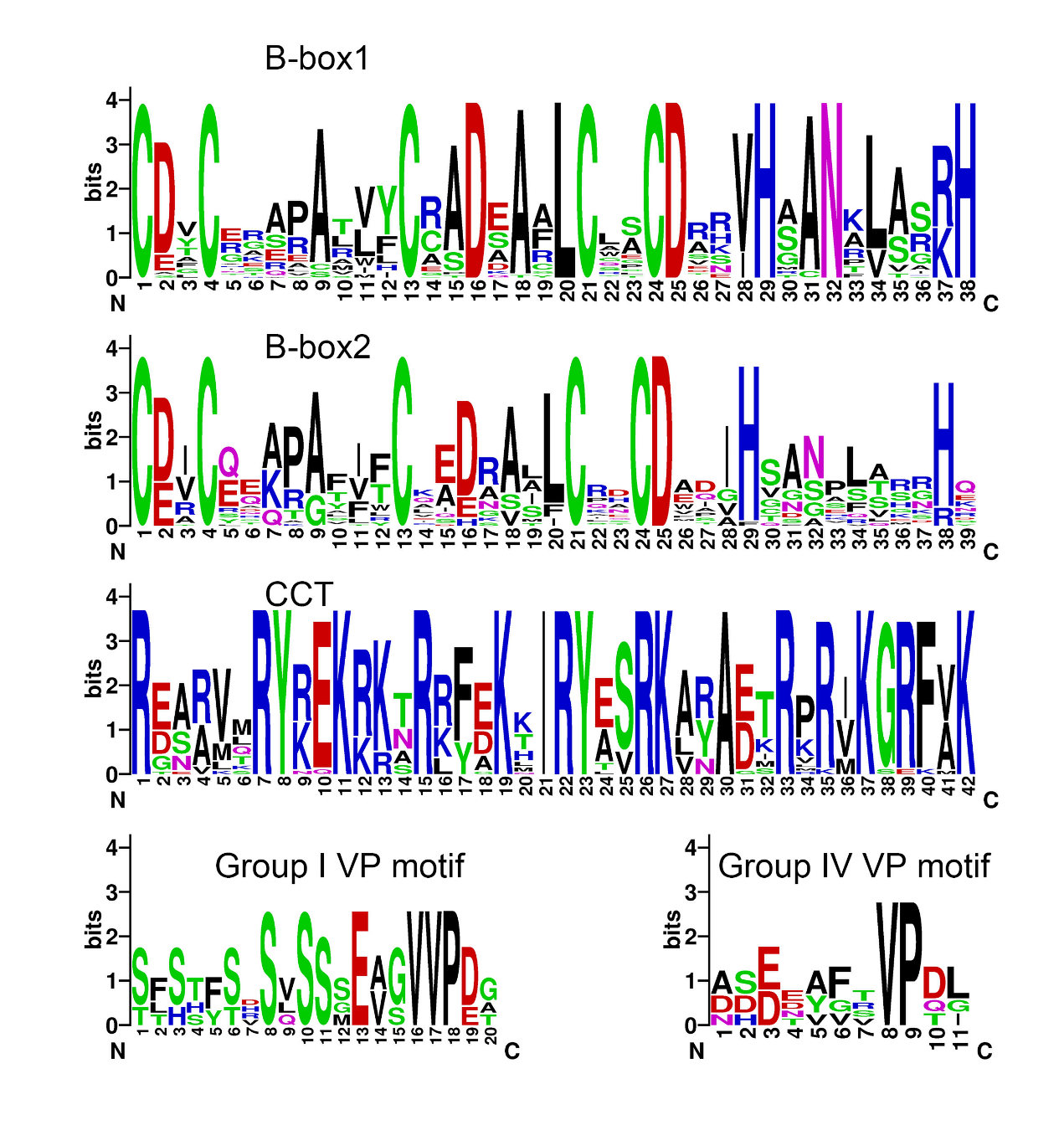


**Supplementary Figure 3 Conserved domains analyses of DrBBX and DaBBX proteins were aligned by a WebLogo program using default parameters.** The B-box1, B-box2, and CCT conserved domain logos were obtained by aligning all the 40 BBXs sequences, 29 group I, II, IV sequences, and 23 group I, II, III sequences form the white Guinea yam and the greater yam, respectively. The group I’s VP motif logo was obtained by aligning the sequences of four group I DrBBXs and four group I DaBBXs. The group IV’s VP motif logo was obtained from DrBBX13, DrBBX14, DrBBX18, DrBBX19, DaBBX12, DaBBX13, DaBBX15, DaBBX15, and DaBBX16.


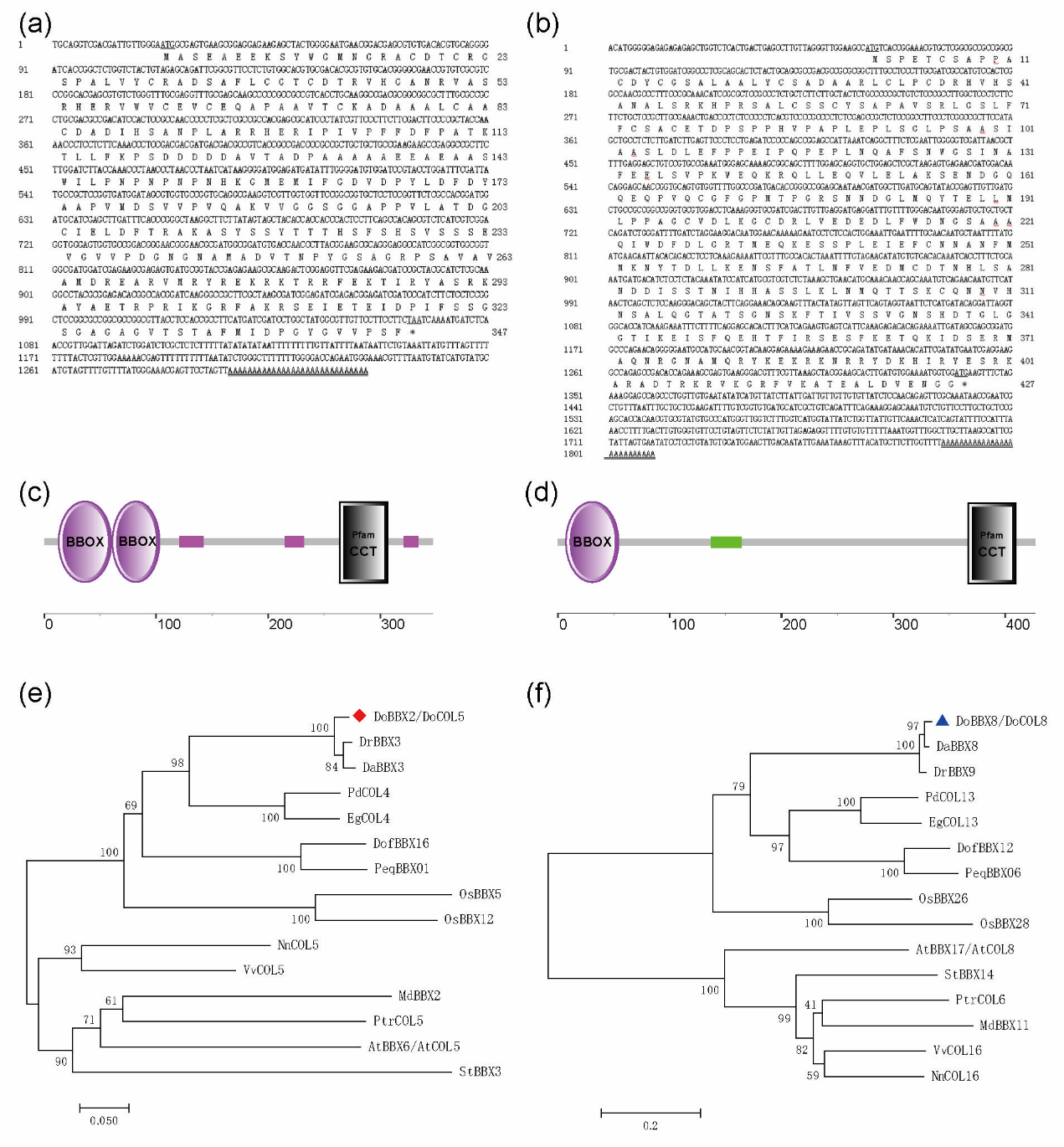


**Supplementary Figure 4 Cloning, domain diagram and phylogenetic analysis of *DoBBX2* and *DoBBX8*.** **(a)** The complete cDNA sequences and amino acid sequences of *DoBBX2* and *DoBBX8*. **(b)** DoBBX6 and DoBBX17 protein domains. **(c)** Phylogenetic analysis of *DoBBX2* and *DoBBX8.* *Do*: *D. opposita* ‘Tiegun’, *Da*: the greater yam (*D. alata*), *Dr*: the white Guinea yam (*D. rotundata*), *Pd*: *Phoenix dactylifera*, *Eg*: *Elaeis guineensis*, *Dof*: *Dendrobium officinale*, *Peq*: *Phalaenopsis equestris*, *Os*: *O. sativa*, *At*: *A. thaliana*, *St*: *Solanum tuberosum*, *Nn*: *Nelumbo nucifera*, *Vv*: *Vitis vinifera*, *Md*: *Malus domestica*, *Ptr*: *Populus trichocarpa*. The red diamond represents DoBBX2/DoCOL5 protein in ‘Tiegun’, and the blue triangle represents DoBBX8/DoCOL8 protein in ‘Tiegun’.


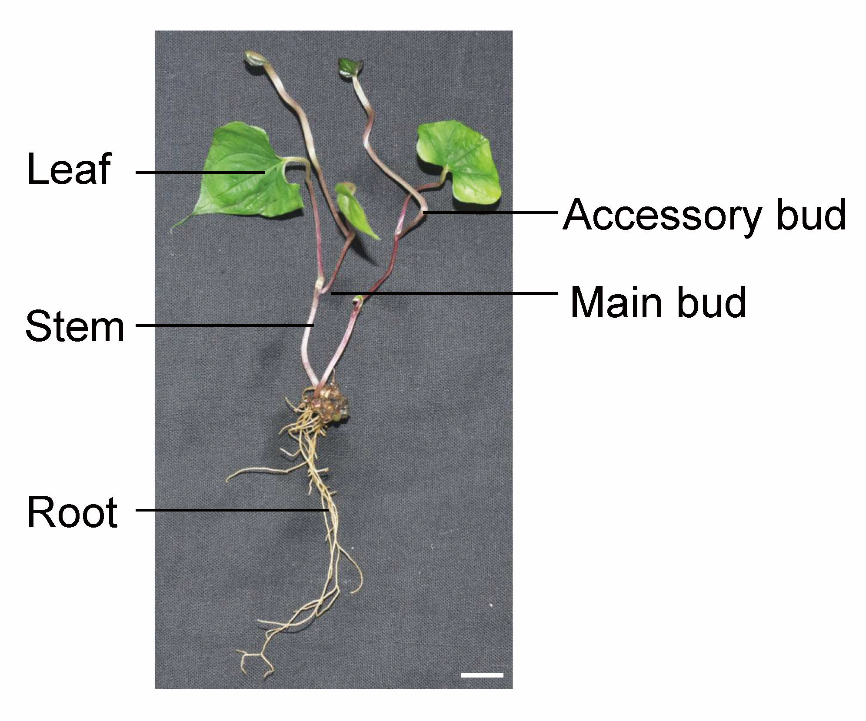


**Supplementary Figure 5** Morphological structure of 45 d-old *D. opposita* ‘Tiegun’ plant. (Bar=1 cm).





**Supplementary Figure 6** The transcript levels of *DoBBX2* and *DoBBX8* in 15-days-old control pants and overexpression plants. **(a)** The quantitative qRT-PCR results. **(b)** The semi quantitative PCR results. Left panel shows the transcript levels of *DoBBX2* gene in 15-days-old control pants (‘E3’) and *DoBBX2* overexpression plants (*DoBBX2*-3, *DoBBX2*-1 and *DoBBX2*-2). The grouping of gels/blots cropped from different parts of the same gel. Right panel shows the transcript levels of *DoBBX8* gene in 15-days-old control pants (‘E3’) and *DoBBX8* overexpression plants (*DoBBX8*-2, *DoBBX8*-3 and *DoBBX8*-1). The grouping of gels/blots cropped from different parts of the same gel. The potato *ELONGATION FACTOR 1 α* (*EF1-α*) gene was used as reference gene for primary data normalization. Error bars indicate standard deviations (SD) from three biological replicates.





**Supplementary Figure 7 Tuber yield per plant in controls and the lines overexpressing *DoBBX2* and *DoBBX8*.** Data are from three independent biological experiments (n = 3 independent biological experiments × 9 growth chambers × 10 individual plants). The red line within the boxplot marks the median (50th percentile) while the black box within the box marks the mean. Significant differences between two means are indicated by Fisher LSD test (P<0.05).
