# Supplementary figures and images for: Identification of BBX gene family and its function in the regulation of microtuber formation in yam
Source: BMC Genomics. 2023 Jun 26;24:354. doi: 10.1186/s12864-023-09406-1 (PMC10291778; doi:10.1186/s12864-023-09406-1)

*DoBBX2*

*DoBBX2*

*DoBBX8*

*DoBBX8*

M E3 3 1 2 E3 3 1 2 E3 2 3 1 E3 2 3 1

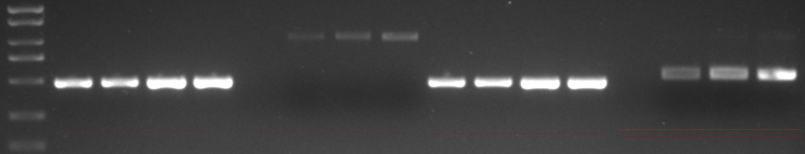

*EF1-α*

*DoBBX2*

*EF1-α*

*DoBBX8*

Supplement: Supplementary file 3 — Additional file 3: Supplementary Info File 1. The semi quantitative PCR result of OE DoBBX2 and DoBBX8 lines. [file 12864_2023_9406_MOESM3_ESM.pdf]
